# Effects of iron-based phosphate binders on mortality and cardiovascular events in patients receiving maintenance dialysis

**DOI:** 10.1038/s41598-023-43177-9

**Published:** 2023-09-25

**Authors:** Akihito Tanaka, Sho Sasaki, Hisashi Noma, Jui Wang, Yoshihiro Onishi, Daijo Inaguma

**Affiliations:** 1 Department of Nephrology, Nagoya University Hospital, Nagoya, Aichi Japan; 2 Section of Education for Clinical Research, Kyoto University Hospital, Kyoto, Japan; 3 Center for Innovative Research for Communities and Clinical Excellence (CiRC2LE), Fukushima Medical University, Fukushima, Japan; 4 Department of Data Science, The Institute of Statistical Mathematics, Tachikawa, Tokyo Japan; 5 College of Public Health, National Taiwan University, Taipei, Taiwan; 6 Institute for Health Outcomes & Process Evaluation Research (iHope International), Kyoto, Japan; 7 Department of Internal Medicine, Fujita Health University Bantane Hospital, Otohbashi 3-6-10, Nakagawa-ku, Nagoya, Aichi 454-8509 Japan

**Keywords:** Kidney, Kidney diseases, Renal replacement therapy

## Abstract

Phosphate binders are the main treatment for hyperphosphatemia in patients with chronic kidney disease, and iron-based phosphate binders have been used with increasing frequency in recent years. This study examined the association of the use of iron-based, rather than non-iron-based, phosphate binders with the incidence of cardiovascular events, in a real-world setting. We used data from a cohort comprising representative adult patients on maintenance hemodialysis in Japan. The exposure of interest was the time-varying use of phosphate binders, classified into “iron-based”, “only non-iron-based”, and “no use”. The primary outcome was a composite of cardiovascular events and all-cause deaths. A marginal structural Cox regression model was used to deal with possible time-dependent confounding. Of the 2247 patients from 58 hemodialysis facilities, iron-based and only non-iron-based phosphate binders were used in 328 (15%) and 1360 (61%), respectively, at baseline. Hazard ratios (95% confidence intervals) for iron-based and non-iron-based phosphate binders versus no use of phosphate binders were 0.35 (0.24, 0.52) and 0.44 (0.33, 0.58), respectively. The hazard ratio for iron-based relative to non-iron-based phosphate binders was 0.81 (0.58, 1.13), which was not statistically significant. Further studies are warranted to elucidate whether the use of iron-based phosphate binders reduces the event rate.

## Introduction

Cardiovascular disease (CVD) is a lethal outcome for patients with chronic kidney disease (CKD), including those with end-stage renal disease (ESRD)^1^. Since hyperphosphatemia is an important cause of CVD in CKD^1–4^, clinical practice guidelines recommended that it be controlled^5,6^.

Phosphate binders are a mainstay in the treatment of hyperphosphatemia in CKD patients. There are three types of phosphate binders: calcium (Ca)-based, iron-based, and others. Although in the past Ca-based phosphate binders were used in high doses, more recently it has become recognized that avoiding overdoses is important, as several studies have reported that Ca-based phosphate binders induce vascular calcification^5–7^. In this context, the most recently launched phosphate binders are iron-based. In Japan, ferric citrate was first used in 2014 and sucroferric oxyhydroxide was first used in 2015.

The relative CVD-preventive effects of iron-based phosphate binders have been directly compared in only one study^8^. In that 2 × 2 randomized controlled trial, lanthanum carbonate and sucroferric oxyhydroxide did not differ on the primary endpoint: suppression of the progression of coronary artery calcification. On the secondary endpoint, in contrast, sucroferric oxyhydroxide may have suppressed that progression^8^. Those results did not rule out the possibility that sucroferric oxyhydroxide, an iron-based phosphate binder, may be cardioprotective. Iron deficiency can develop in CKD patients during treatment of renal anemia, and it can lead to erythropoiesis stimulating agent (ESA) resistance. Indeed, guidelines^9,10^ recommend iron supplementation based on serum ferritin and transferrin saturation. Since renal anemia is a risk factor for CVD, iron-based phosphate binders may reduce cardiovascular risk through stabilization of iron dynamics. Therefore, iron-based phosphate binders that can simultaneously treat hyperphosphatemia, iron kinetics, and anemia are expected to be beneficial.

To date, there have been no studies comparing the use of iron-based versus iron-free phosphate binders on the risk of CVD and of all-cause death. We therefore examined the association of the use of iron-based, rather than non-iron-based, phosphate binders with a composite of cardiovascular events and all-cause mortality in maintenance hemodialysis patients, using data from the Japanese part of the international Dialysis Outcomes and Practice Patterns Study (J-DOPPS).

## Materials and methods

### Study population

The Dialysis Outcomes and Practice Patterns Study (DOPPS) is a prospective, international cohort study of representative samples of hemodialysis patients. In the DOPPS, nationally-representative dialysis facilities in each country are enrolled and hemodialysis patients are selected randomly from them. The DOPPS design has been reported in detail^11^. Here we used data from J-DOPPS Phase 6 that were collected at 58 facilities between 2015 and 2018 as part of the DOPPS. Informed consent was obtained from all subjects and/or their legal guardian(s). The study complied with the Declaration of Helsinki, and was approved by the Ethics Committee of Fujita Health University (approval number: HM22-213).

The target population was adult hemodialysis patients. We excluded patients who met one or more of the following three criteria: having been on dialysis for fewer than 30 days, having a serum ferritin level of 800 ng/ml or higher at the start of the observation period, and undergoing dialysis less often than twice per week throughout the observation period.

### Exposures

The exposure of primary interest was the use of iron-based phosphate binders in comparison with the use of non-iron-based phosphate binders. The former was defined as the use of iron-based phosphate binders either alone or in combination with non-iron-based phosphate binders, while the latter was any use of phosphate binders but not iron-based phosphate binders, where iron-based phosphate binders included ferric citrate and sucroferric oxyhydroxide, and non-iron-based phosphate binders included calcium carbonate, sevelamer hydrochloride, and lanthanum carbonate.

Since physicians’ decisions to prescribe these drugs in clinical practice vary with time, depending on the patient’s laboratory data and the prescription status of other drugs, the prescribing information for phosphate binders was handled as a time-dependent variable. For time-dependent analyses mentioned later, we set three levels of the use of phosphate binders: “iron-based”, “only non-iron-based”, and “no use”. The third level, “no use”, was necessary because there are inevitably some periods without any use of phosphate binders. The unit period of exposure was set at four months, as the study variables were measured every four months in the DOPPS. The level of use of phosphate binders was set as status at the beginning of each unit period.

### Outcomes

The main outcome was a composite of CVD events and all-cause deaths. CVD events comprised stroke, cardiac arrest, angina pectoris, cerebral hemorrhage, congestive heart disease, pulmonary edema, transient ischemic attack, acute myocardial infarction, and hospitalizations due to coronary angioplasty or coronary artery bypass grafting. As a secondary outcome, all-cause deaths alone were examined. Patients meeting any of the following conditions were censored: transfer to another facility, transplantation, and modality switch (peritoneal dialysis, home dialysis, and discontinuation of dialysis).

### Covariates

As potential confounders, we considered patient characteristics at the start of the observation period (age, gender, hemodialysis vintage, presence of diabetes mellitus, previous CVD, use of renin-angiotensin system inhibitors and beta-blockers) and clinical status (data collected every 4 months, comprising hemoglobin, serum CRP, serum calcium, serum ferritin, serum albumin, serum phosphorus, serum potassium, on-dialysis weight loss, iron saturation ratio, and serum parathyroid hormone (PTH), as well as prescriptions of iron drugs other than phosphate binders, vitamin D analogs, and calcimimetics).

### Statistical analysis

Continuous data are summarized as means and standard deviations (SD) or medians and interquartile ranges (IQRs). Categorical data are summarized as numbers and proportions. Crude incidence rates were computed as summary measures of incidence frequencies. Values of hemoglobin, transferrin saturation, and ferritin were summarized by time-dependent levels of the use of phosphate binders, to describe iron load throughout the observation period.

Marginal structural models (MSMs) have been used to adjust for time-dependent confounders^12,13^. We used a marginal structural Cox regression model to assess the effects of the use of “iron-based” and “only non-iron-based” phosphate binders. The time-varying weights were calculated from the inverse of the probability of having the history of the use of phosphate binders that a patient actually had during each 4-month period of observation. These probabilities were predicted from a pooled trinomial logistic regression model. In that model, the dependent variable was the use of phosphate binders, and the independent variables were the potential confounders listed above. We used stabilized weights in the MSM^12,14^. Similarly, we adjusted for censoring by using the inverse probability of censoring weighting (IPCW)^12^. The censoring weights were calculated from the following predictors to account for loss to follow-up: age, gender, dialysis vintage, activities of daily living (eating/mobility), marital status, living conditions (living alone or not), blood pressure, on-dialysis weight loss, incidence of malignancy, incidences of hospitalization due to CVD, dementia, stroke, or cerebral hemorrhage, as well as serum concentration of albumin, CRP, potassium, and phosphate. Weighted Cox regression with robust variance was used to estimate hazard ratios (HRs) and 95% confidence intervals (95% CI) with “no use” as a reference. Finally, the effect of the use of “iron-based” versus “only non-iron-based” phosphate binders was computed. We used the robust standard error estimator, and thus the confidence intervals for the HRs should be correct even if there are cluster effects caused by facilities.

Missing data were imputed using a multivariate imputation by chained equations (MICE) algorithm. The results for 200 imputed datasets were combined using Rubin’s rule.

We used subgroup analyses to explore the effect heterogeneity of the use of “iron-based” versus “only non-iron-based” phosphate binders. The variables for subgrouping were age, sex, and comorbid CVD. We estimated adjusted hazard ratios using the models mentioned above.

Statistical analyses were done using R version 4.1.0 (R Foundation for Statistical Computing, Vienna, Austria). All p values shown are two-sided, and p values less than 0.05 were taken as indicators of statistical significance.

## Results

### Patient characteristics

Of the 2247 patients who met the eligibility criteria, 1527 (68%) were male, 985 (44%) had comorbid diabetes mellitus, and 1321 (59%) had comorbid CVD. The mean age of the participants was 66.2 years (SD 12.0), and the mean dialysis history was 3.8 years (IQR 0.9, 9.6). “Iron-based” and “only non-iron-based” phosphate binders were used in 328 (15%) and 1360 (61%) participants at baseline, respectively. Baseline characteristics are shown in Table 1. Compared with the non-iron-based only group, patients in the iron-based group were younger, had more diabetes, higher transferrin saturation, and higher ferritin, and received fewer intravenous or oral iron drugs.

**Table 1:** Patient characteristics at baseline.

Items	All subjects (N = 2247)	Stratified by the use of phosphate binders at baseline		
		Iron-based (N = 328)	Only non-iron-based (N = 1360)	None (N = 559)
Age	66 (12)	64 (12)	65 (12)	70 (12.0)
Male sex	68%	67%	67%	70%
Hemodialysis vintage	3.8 [0.9,9.6]	3.5 [0.6,7.7]	5.1 [1.8,11.7]	1.9 [0.3,6.3]
Comorbidities				
Diabetes mellitus	44%	46%	41%	50%
Cardiovascular disease	59%	58%	58%	62%
Laboratory				
Hemoglobin (g/dL)	10.9 (1.2)	11.2 (1.3)	11.0 (1.2)	10.7 (1.3)
Albumin (g/dL)	3.6 (0.4)	3.6 (0.4)	3.6 (0.4)	3.5 (0.5)
C-reactive protein (mg/dL)	0.1 [0.1–0.4]	0.1 [0.1–0,3]	0.1 [0.1–0.4]	0.2 [0.1–0.6]
Potassium (mEq/L)	4.7 (0.7)	4.8 (0.7)	4.8 (0.7)	4.5 (0.7)
Calcium (mg/dL)	8.7 (0.7)	8.8 (0.8)	8.8 (0.7)	8.6 (0.7)
Phosphate (mg/dL)	5.3 (1.4)	5.6 (1.5)	5.3 (1.4)	4.9 (1.3)
iPTH (pg/ml)	132 [74–225]	140 [73–236]	126 [72–222]	138 [82–225]
Transferrin saturation (%)	23 [16–31]	26 [18–36]	23 [16–31]	21 [15–30]
Ferritin (ng/ml)	73 [35–141]	92 [42–171]	67 [31–133]	77 [36–148]
Living alone	15%	12%	16%	14%
ADL independence	88%	90%	90%	81%
Medication				
RAASi	50%	59%	50%	45%
Beta-blockers	11%	9%	11%	11%
ESA	88%	87%	88%	86%
Iron	38%	24%	42%	37%
Vitamin D analogs	70%	74%	74%	57%
Calcimimetics	22%	24%	27%	8%

*iPTH* intact parathyroid hormone, *RAASi* renin–angiotensin–aldosterone system inhibitors, *ESA* erythropoietin stimulating agents.

Values are mean (standard deviation), median [interquartile range], or proportion.

**Figure 1:**
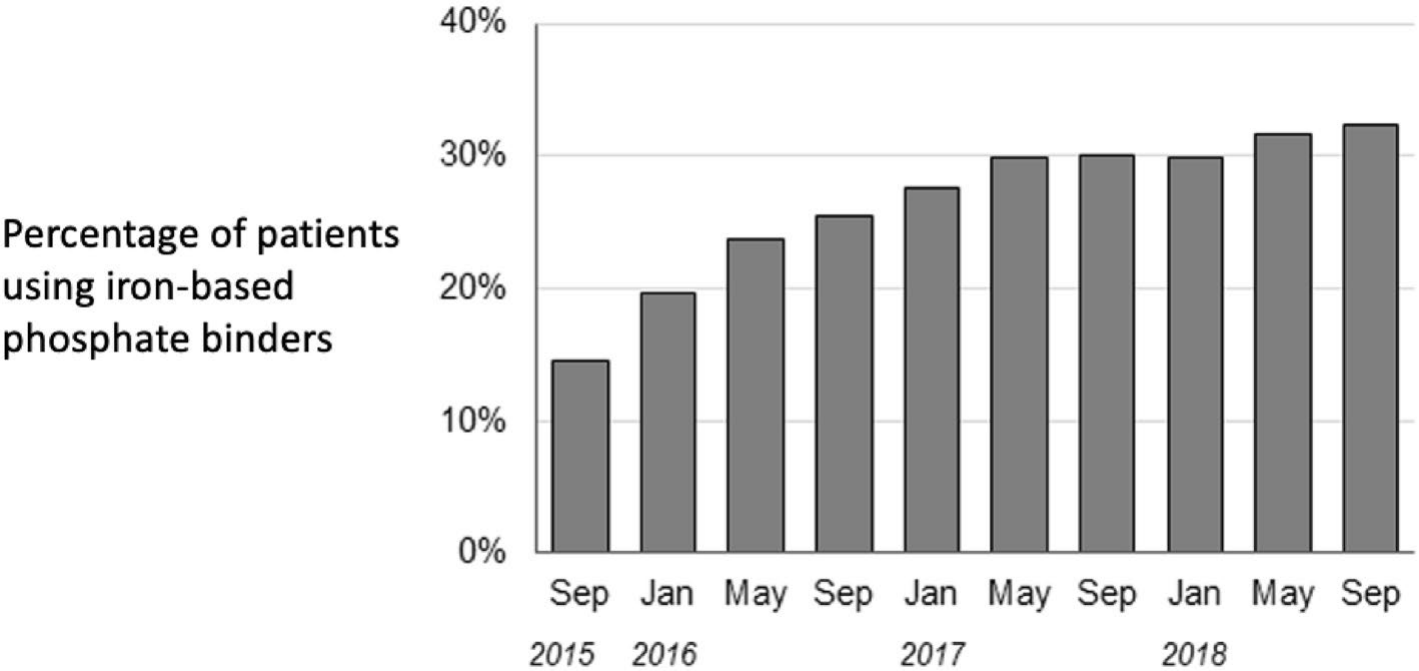
Use of phosphate binders during the study period.

Figure 1 shows changes over time in the percentage of patients to whom iron-based phosphate binders were prescribed. Prescriptions of these drugs increased from 15 to 32% during this study’s observation period.

### Association between use of different types of phosphate binders and outcomes

The median follow-up period was 2.67 years (IQR 1.33, 2.68). The crude incidence rates (/100 person-years) of the composite outcome were 5.10, 6.43, and 16.6, for the uses of “iron-based” phosphate binders, “only non-iron-based” phosphate binders, and “no use” of phosphate binders, respectively. A similar relationship was observed in the crude incidence of all-cause deaths (Table 2).

**Table 2:** Crude incidence rates of outcomes by time-dependent exposure levels.

	Use of phosphate binders (time-dependent)			Total
	Iron-based	Only non-iron-based	No use	
CVD events and all-cause deaths				
Cumulative exposure period [person-years]	1157	2504	687	4348
Number of events	59	161	114	334
Incidence rate [/100 person-years]	5.1	6.4	16.6	7.7
All-cause deaths				
Cumulative exposure period [person-years]	1192	2,569	712	4472
Number of events	36	99	92	227
Incidence rate [/100 person-years]	3.0	3.9	12.9	5.1

*CVD* cardiovascular disease.

**Figure 2:**
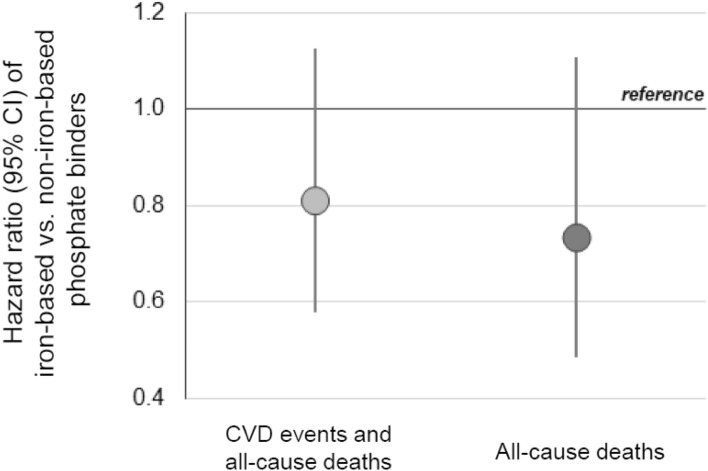
Adjusted hazard ratios of clinical outcomes for the use of iron-based phosphate binders in comparison with those for only non-iron-based phosphate binders. *CVD* cardiovascular disease.

The adjusted HRs for the use of phosphate binders are shown in Table 3. The hazard for the composite outcome was lowest for the use of “iron-based” phosphate binders. The hazard was also significantly lower for “only non-iron-based” phosphate binders than for “no use”. All-cause deaths were also significantly lower for both “iron-based” and “only non-iron-based” binders, and the effect size was greater for “iron-based” binders. The HRs for “iron-based” binders relative to “only non-iron-based” binders were low: 0.81 (95% CI 0.58, 1.13; p = 0.21) and 0.73 (95% CI 0.49, 1.11; p = 0.14), respectively, for the composite outcome and for all-cause deaths, neither of which was statistically significant (Fig. 2). No apparent heterogeneity of the HRs was noted between subgroups by age, sex, and comorbid CVD (Supplementary Table S1 online).

**Table 3:** Adjusted hazard ratios for time-dependent exposures.

Exposure	CVD events and all-cause deaths			All-cause deaths		
	Hazard ratio (95% CI)		p value	Hazard ratio (95% CI)		p value
Use of phosphate binder						
Iron-based	0.35	(0.24, 0.52)	< 0.001	0.25	(0.16, 0.39)	< 0.001
Only non-iron-based	0.44	(0.33, 0.58)	< 0.001	0.34	(0.24, 0.47)	< 0.001
No use	reference			reference		

*CVD* cardiovascular disease.

### Iron-related laboratory values during the study

Iron-related laboratory values by time-dependent levels of the use of phosphate binders are shown in Table 4. The highest values were observed in the use of “iron-based phosphate binder.”

**Table 4:** Summary of iron-related laboratory values by time-dependent exposures.

Items	Use of phosphate binders (time-dependent)		
	Iron-based	Only non-iron-based	None
Hemoglobin (g/dL)	11.3 (1.3)	11.0 (1.2)	10.6 (1.4)
Transferrin saturation (%)	28 [21–36]	23 [16–31]	24 [16–31]
Ferritin (ng/ml)	104 [54–185]	65 [32–134]	74 [36–147]

Values are mean (standard deviation) or median [interquartile range].

## Discussion

### Summary of the results

The patients who received iron-based phosphate binders had the lowest incidence of CVD events and all-cause deaths. While the difference between the HRs for the “iron-based” and the “only non-iron-based” groups was not statistically significant, one can see that iron-based phosphate binders were estimated to have reduced the incidence of the primary outcome (the composite of CVD events and all-cause deaths) by approximately 20% compared to the non-iron-based phosphate binders. When the secondary outcome, all-cause deaths, was compared similarly, iron-based phosphate binders were estimated to have reduced the incidence by approximately 25%. Effect heterogeneities were not evident in the subgroups examined. To our knowledge, the present study is the first to show how the use of iron-based phosphate binders is associated with important clinical outcomes.

### Comparison with previous reports on the association of phosphate binders with CVD events and mortality

Physicians have hesitated to prescribe calcium-based phosphate binders in large quantities because of concerns that calcium loading may induce vascular calcification^5–7^. However, a recent study comparing the mortality of patients receiving lanthanum carbonate with the mortality of those receiving a calcium-based phosphate binder showed no advantage of lanthanum carbonate^15^. According to a recent meta-analysis of randomized controlled trials done in patients with predialysis CKD^16^, calcium-free phosphate binders significantly reduced serum phosphate levels and urinary phosphate excretion compared with placebo, but there was no evident advantage regarding the risk of CVD. In patients receiving hemodialysis, an observational study^17^ reported that sevelamer initiation was associated with a lower risk of mortality, and speculated that the mechanisms involved were serum phosphorus control and reduced calcium loading. In addition to these mechanisms, iron-based phosphate binders modulate iron metabolism, and have been suggested to inhibit the progression of coronary artery calcification in patients with ESRD^8^. However, evidence from large clinical studies with mortality and CVD events as outcomes is lacking. Hence, the present study is important as it reports on CVD events and mortality associated with the use of iron-based phosphate binders.

### Favorable effects of iron-based phosphate binders via iron supplementation

For patients with diseases other than CKD, iron supplementation during iron deficiency can reduce heart-failure events^18,19^. In addition, iron is involved in hematopoiesis and iron supplementation can be used to treat anemia when iron is deficient. When patients on dialysis have severe anemia, their prognosis is poor^20,21^ and correction of severe anemia is recommended in Japan’s guidelines^10^. Furthermore, in CKD patients with or without anemia, iron deficiency on its own is associated with mortality^22^. In addition to the importance of avoiding iron deficiency, observational studies in patients at the induction phase of dialysis suggest that simultaneous management of anemia and iron dynamics, in addition to phosphate management, may lead to a better prognosis^23^. Thus, eliminating iron deficiency is expected to increase life expectancy, either by lowering the risk of anemia, or by lowering the risk of heart failure unrelated to anemia. While iron deficiency should be avoided, there is a concern that iron excess to the point of iron deposition in organs may decrease life expectancy. In the present study, the highest hemoglobin level was observed in the “iron-based phosphate binder” group. That group also had the highest transferrin saturation levels and ferritin levels, but they were within the recommended range, indicating that there was no iron overload (based on Japan’s guidelines, which recommend that transferrin saturation should be at least 20% and ferritin should be from 50 or 100 to 300 ng/ml^10^). Thus, in addition to their primary purpose of phosphate management, iron-based phosphate binders may have reduced the risk of CVD events and mortality, either directly (cardioprotective effect) or indirectly by ameliorating anemia, since they can function as iron supplements.

### Strengths

First, unlike a previous report^8^ that was limited to the evaluation of surrogate markers such as coronary artery calcification, in the present study we measured important clinical outcomes: CVD events and all-cause deaths. Second, this study was based on data from routine clinical practice. That is important because in daily clinical practice keeping serum phosphate levels within the target range by using only one phosphate binder is difficult, so two or more phosphate binders are often used together. Thus, this study’s results should be close to those that can be expected in clinical practice. Third, to address the treatment-outcome interrelationship (time-dependent confounding) statistically, we used MSMs. Prescriptions for phosphate binders can vary with phosphate levels and other factors. If phosphate levels decrease because of phosphate-binder use, prescriptions may be changed accordingly. Moreover, as shown in Fig. 1, the percentage of patients in whom iron-based phosphate binders were prescribed increased during the study period. Because of those changes over time, the data must be analyzed with time-dependent confounding taken into account, as we did here. This contributes to the relevance of these results for clinical practice. Fourth, the data came from a representative sample of dialysis patients in Japan, with no regional bias.

### Limitations

One limitation may be the sample size. The confidence intervals (for example, in Fig. 2) would almost certainly have been narrower if there had been more patients in the study, and with 2247 participants the study might have lacked sufficient statistical power. We hope that future studies will include larger samples. Also, because this was an observational study, the possibility of residual confounding cannot be ruled out. A higher percentage of patients who received no phosphate binders had a history of diabetes or CVD, so for them the likelihood of an adverse outcome might have been high. However, the baseline characteristics of the patients who received iron-based phosphate binders were not very different from those of the patients who only received non-iron-based phosphate binders, which suggests that substantial bias in the comparison of the uses of different phosphate binders was unlikely.

## Conclusion

Compared with patients receiving iron-free phosphate binders, those receiving iron-based phosphate binders were less likely to have the combined outcome of CVD events and all-cause deaths, but the difference was not statistically significant.

### Supplementary Information

Supplementary Table S1.

## Data Availability

No data are available. The data that support the findings of this study are available from the Arbor Research Collaborative for Health. Restrictions apply to the availability of those data. For the current study, those data were used under license, and thus they are not publicly available. However, requests for data can be sent to Arbor Research via their website (http://www.arborresearch.org/AboutUs/ContactUs.aspx). Those who would like to discuss obtaining the data may contact Yoshihiro Onishi (onishi@i-hope.jp).
