# Supplementary material for: Effects of iron-based phosphate binders on mortality and cardiovascular events in patients receiving maintenance dialysis
Source: Sci Rep. 2023 Sep 25;13:16051. doi: 10.1038/s41598-023-43177-9 (PMC10520071; doi:10.1038/s41598-023-43177-9)
Supplement: Supplementary file 1 — Supplementary Table S1. [file 41598_2023_43177_MOESM1_ESM.pdf]

Supplementary Table S1. Adjusted hazard ratios of clinical outcomes for the use of “iron-based” phosphate binders in comparison with those for “only non-iron-based” phosphate binders, stratified by patients’ characteristics.

Subgroups		n	CVD events and all-cause deaths		All-cause deaths	
			Hazard ratio (95% CI)	p value	Hazard ratio (95% CI)	p value
Age	< 65 y	913	0.66 (0.30, 1.42)	0.29	0.65 (0.30, 1.42)	0.28
	≥ 65y	1434	0.86 (0.52, 1.40)	0.54	0.82 (0.51, 1.34)	0.43
Sex	Male	1594	0.68 (0.41, 1.12)	0.13	0.68 (0.41, 1.10)	0.12
	Female	756	0.99 (0.47, 2.12)	0.99	0.93 (0.44, 1.96)	0.84
Comorbid CVD	No	964	0.87 (0.35, 2.18)	0.76	0.84 (0.33, 2.11)	0.71
	Yes	1359	0.73 (0.46, 1.16)	0.19	0.71 (0.45, 1.13)	0.15

CVD, cardiovascular disease; CI, confidence intereval
